# Acacetin inhibited non-small-cell lung cancer (NSCLC) cell growth via upregulating miR-34a in vitro and in vivo

**DOI:** 10.1038/s41598-024-52896-6

**Published:** 2024-01-29

**Authors:** Jing Li, Xianmei Zhong, Yueshui Zhao, Jing Shen, Zhangang Xiao, Chalermchai Pilapong

**Affiliations:** 1 Laboratory of BioMolecular Imaging, Molecular and Cellular Biology, Department of Radiologic Technology, Faculty of Associated Medical Sciences, Chiang Mai University, Chiang Mai, 50200 Thailand; 2 Department of Oncology and Hematology, The Affiliated Traditional Chinese Medicine Hospital, Southwest Medical University, Luzhou, 646000 China; 3 Laboratory of Molecular Pharmacology, Department of Pharmacology, School of Pharmacy, Southwest Medical University, Luzhou, 646000 China; 4 Cell Therapy & Cell Drugs of Luzhou Key Laboratory, Southwest Medical University, Luzhou, 646000 China; 5 South Sichuan Institute of Translational Medicine, Luzhou, 646000 China; 6 Department of Pharmacy, People’s Hospital of Nanbu County, Nanchong, 637300 China

**Keywords:** Cancer, Lung cancer, Non-small-cell lung cancer

## Abstract

Acacetin, one of the flavonoid compounds, is a natural product found in various plants, including *Silver birch*, and *Damiana*. Previous studies showed that acacetin has anti-cancer effects on many kinds of cancer cells, however, the role of and the mechanisms of actions of acacetin on non-small cell lung cancer (NSCLC) cells is still not fully understood. Herein, we found that, in vitro, acacetin inhibited the proliferation, invasion, and migration of NSCLC cells, A549 and H460, in a dose-dependent manner. Meanwhile, flow cytometry assay results showed that acacetin induced G2/M phase cell cycle arrest, and apoptosis of NSCLC cells. In vivo, acacetin suppressed tumor formation of A549-xenografted nude mice model with no obvious toxicities. Western blotting results showed that the protein levels of cell cycle-related proteins cyclin B1, cyclin D, and anti-apoptotic protein Bcl-2 had decreased, while the apoptosis-related protein Bak had increased both in NSCLC cells and in A549-xenografted tumor tissues. For investigating the molecular mechanism behind the biological effects of acacetin on NSCLC, we found that acacetin induced the expression levels of tumor suppressor p53 both in vitro and in vivo. MicroRNA, miR-34a, the direct target of p53, has been shown anti-NSCLC proliferation effects by suppressing the expression of its target gene programmed death ligand 1 (PD-L1). We found that acacetin upregulated the expression levels of miR-34a, and downregulated the expression levels of PD-L1 of NSCLC cells in vitro and of tumors in vivo. In vitro, knockdown p53 expression by siRNAs reversed the induction effects of acacetin on miR34a expression and abolished the inhibitory activity of acacetin on NSCLC cell proliferation. Furthermore, using agomir and antagomir to overexpress and suppress the expression miR-34a in NSCLC cells was also examined. We found that miR-34a agomir showed similar effects as acacetin on A549 cells, while miR-34a antagomir could partially or completely reverse acacetin’s effects on A549 cells. In vivo, intratumor injection of miR-34a antagomir could drastically suppress the anti-tumor formation effects of acacetin in A549-xenografted nude mice. Overall, our results showed that acacetin inhibits cell proliferation and induces cell apoptosis of NSCLC cells by regulating miR-34a.

## Introduction

Non-small cell lung cancer (NSCLC) is the most common type of lung cancer that accounts for approximately 85% of all lung cancer cases and is responsible for a significant number of cancer-related deaths worldwide each year. Traditional therapy methods for lung cancer are surgery, radiation therapy, chemotherapy, and targeted drug therapy. However, these kinds of therapies are not very promising for some patients with advanced lung cancers.

Recently, researchers showed major interest in plant derived natural compounds (also called phytochemicals), such as flavonoids, for cancer prevention and treatment, as these compounds showed high bioavailability to kill cancer cells, and lower adverse effects^1,2^. Acacetin, a kind of flavonoid compound, has been shown to have anti-inflammatory, and anti-infectious effects of different pathophysiological conditions^3^, while also showing anti-cancer properties in various cancer cells including breast, liver, prostate, and colon cancer cells. Acacetin inhibited cancer cell proliferation by regulating and inducing apoptosis via caspase cascades^4^ or by stimulating reactive oxygen species (ROS) formation^5^. Further studies showed that acacetin could induce cancer cell arrest in the G1 phase of liver and breast cancer cells^6^ or arrest in the G2/M phase of colon cancer cells^7^. In addition, acacetin could inhibit the invasion and migration of cancer cells via regulating PI3K/Akt/Snail pathway of gastric cancer cells^8^, or by regulating p38 MAPK signaling pathway of prostate cancer cells^9^. For lung cancer cells, acacetin could induce cell cycle arrest, apoptosis, invasion, and migration of NSCLC cells in vitro^10^. However, the underlying mechanisms are still not clearly understood, and questions regarding whether acacetin can actually inhibit tumor formation in vivo remain unanswered.

microRNAs, a group of endogenous non-coding small RNAs, serve as tumor activators or suppressors. One of the potential microRNAs for NSCLC therapy is miR-34a. miR-34a was downregulated in NSCLC tissues and cell lines^5^. miR-34a acts as a tumor-suppressor gene by dysregulating the expression levels of many key oncogenic target genes related to cancer cell proliferation, invasion, and anti-apoptosis. Low expression of miR-34a in NSCLC tissues was related to unfavorable clinical outcomes^6,7^. It appears that miR-34a inhibits NSCLC cell growth by targeting Sirtuin (SIRT) 6^11^, LIM domain containing 2 (LIMD2)^12^, or programmed death ligand 1 (PD-L1)^13^, and it also suppresses the invasion of NSCLC cells by targeting LIMD2^12^. Polyphenols, including resveratrol, honokiol, or polyphenol extracted from pomegranate rinds, have been found to demonstrate anti-proliferative effects by upregulating miR-34a levels^14^. However, whether acacetin regulates miR-34a expression in NSCLC cells is still not known.

In this present study, by using in vitro NSCLC cells and in vivo NSCLC cell-xenografted nude mice model, we investigated the role of and the potential mechanism of acacetin on the proliferation/viability, invasion, and migration of NSCLC cells, and investigate whether miR-34a participates in these processes.

## Results

### Acacetin inhibits cell proliferation, migration, and invasion of NSCLC cells in vitro

**Figure 1:**
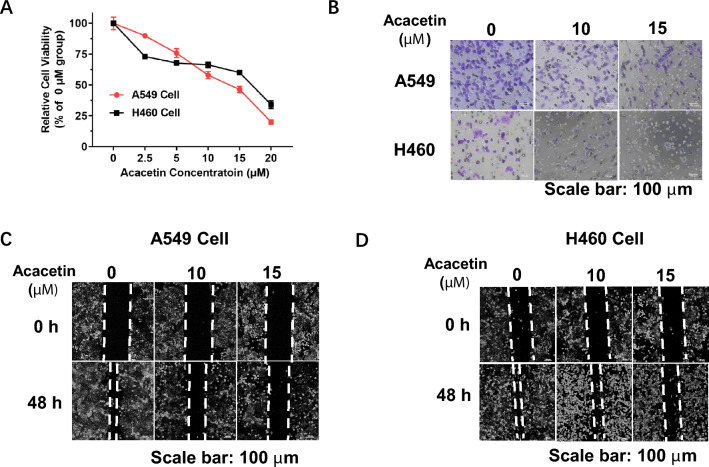
Acacetin inhibits cell proliferation, invasion, and migration of NSCLC cells. (**A**) NSCLC cells, H460 and A549, were treated with different doses of acacetin for 48 h, cell viability was then detected by MTT assay (n = 6). (**B**) Cell invasion assay of H460 and A549 cells which were treated with 0, 10, and 15 μM of acacetin for 48 h (n = 3). (**C**, **D**) Migration assay of H460 and A549 cells, which were treated with 0, 10, and 15 μM of acacetin for 48 h (n = 3).

To investigate the effects of acacetin on NSCLC cells, firstly, we checked whether acacetin regulates the proliferation of NSCLC cells. A549 and H460 cells were treated with different concentrations of acacetin (0–20 μM) for 48 h, then detected the cell viability by MTT assay. As shown in Fig. 1A, acacetin dramatically inhibited both NSCLC cells growth in a dose-dependent manner, while the inhibition effect was more obvious on A549 cells than on H460 cells.

The migratory and invasive activities are important for the metastases of NSCLC cells. Next, we evaluated the effects of acacetin on the migration and invasion of NSCLC cells. When A549 and H460 cells were treated with 10 and 15 μM of acacetin for 48 h, the invasion ability (Fig. 1B) and migration ability (Fig. 1C,D) were significantly inhibited.

### Acacetin induces G2/M phase cell cycle arrest and apoptosis of NSCLC cells.

**Figure 2:**
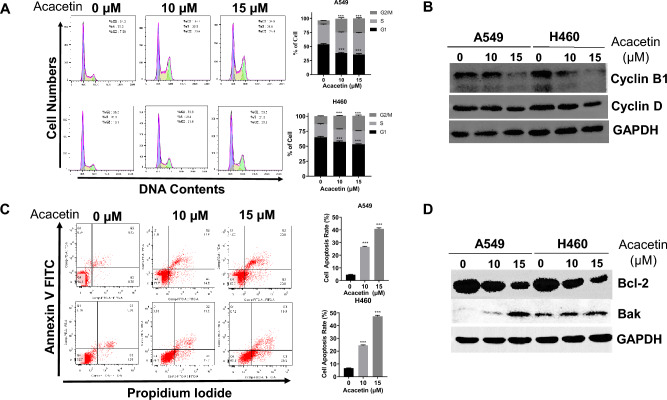
Acacetin induces G2/M phase cell cycle arrest and apoptosis of NSCLC cells. A549 and H460 cells were treated 0, 10, and 15 μM of acacetin for 48 h, (**A**) Cell cycle phases were analyzed by flow cytometry, and the histogram graphs shown are representative of three parallel and independent experiments (***, *p* < 0.001, compared with 0 μM group). (**B**) Western blotting results for cell cycle-related proteins, cyclin B1 and cyclin D, of cells (GAPDH was used as internal control for western blotting). (**C**) Cells were stained with Annexin V-FITC and PI, and the apoptotic rate was then analyzed. The histogram graphs shown are representative of three parallel and independent experiments (***, *p* < 0.001, compared with 0 μM group). (**D**) Western blotting results for apoptosis-related proteins, Bcl-2 and Bak, of cells (GAPDH was used as internal control for western blotting).

As cell proliferation is controlled by cell cycle progression, next, we checked whether acacetin regulates the cell cycle of NSCLC cells. A549 and H460 cells were treated with acacetin (10 and 15 μM) for 48 h, cell cycle was then analyzed by flow cytometry. The results showed that acacetin induced an increase in G2/M phase of both NSCLC cells, indicating cell cycle arrest occurring in G2/M phase (Fig. 2A). Cyclin B1 are key regulator of cell cycle transition from G2 phase to M (mitosis) phase^15^, and we also checked the protein levels of cyclin B1. As shown in Fig. 2B, acacetin significantly inhibited the protein levels of cyclin B1 in a dose-dependent manner in both A549 and H460 cells. Moreover, the expression levels of cyclin D had also decreased after acacetin treatment (Fig. 2B).

When we performed the cell viability assay, we found dead cells in the acacetin-treated groups, as apoptosis is one of the major types of chemotherapeutic drugs-induced cancer cell deaths. Studies have shown that G2/M phase cell cycle arrest is a major factor that induces cancer cell apoptosis^16^. We next evaluated whether acacetin induces apoptosis of NSCLC cells. A549 and H460 cells were treated with acacetin (10 and 15 μM) for 48 h and apoptotic rate was analyzed by flow cytometry using Annexin-V and PI staining. As shown in Fig. 2C, acacetin dramatically induced apoptosis in both A549 and H460 cells, compared with the control (0 μM) group (*p* < 0.001). Meanwhile, compared with 10 μM acacetin-treatment group, the levels of both pro-apoptotic cells and late-stage apoptotic cells had dramatically increased in 15 μM acacetin-treatment groups (*p* < 0.001) of A549 and H460 cells.

Moreover, the levels of anti-apoptosis protein Bcl-2 had decreased, whereas the protein levels of pro-apoptotic molecule Bak had increased after acacetin treatment in both A549 and H460 cells (Fig. 2D).

### Acacetin inhibited tumor formation of NSCLC-xenografted mice model

Next, we investigated the in vivo effects of acacetin on NSCLC tumors. BALB/c athymic nude mice were injected with A549 cells for 10 days when tumor size is approximately 50 mm^3^, followed by the i.p. administration of Acacetin at (0, 10, or 20 mg/kg body weight) every other day for 30 days.

At the end of the experiment, we firstly analyzed whether acacetin showed potential toxicities in animals. Serum levels of alanine aminotransferase (ALT) and aspartate aminotransferase (AST) have been used as major biomarkers for liver damage, especially as biomarkers for substances-induced liver toxicity^17,18^. We found that 10 mg/kg acacetin showed no effect, while 20 mg/kg acacetin significantly reduced the serum levels of ALT and AST (Supplementary Fig. 1A). We also collected the major organs (including the liver, lung, kidney, and spleen) for analyses. There were no significant difference between the weights of the liver, lung, and kidney of each group (Supplementary Fig. 1B). H&E staining results also showed no obvious change in the structures of the liver, lung, kidney, and spleen of acacetin-treated mice, compared with control group (Supplementary Fig. 1C). The above data suggest that there were no significant side effects when the mice were treated with 10 or 20 mg/kg of acacetin.

**Figure 3:**
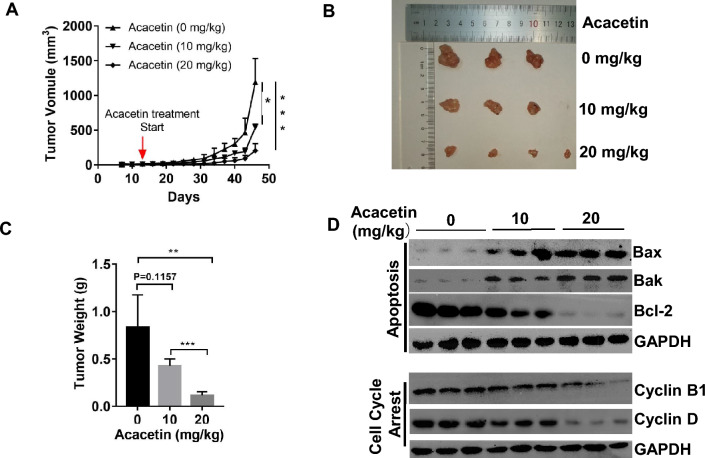
Acacetin inhibited tumor formation of NSCLC xenograft mice. (**A**) The changes of tumor volume. Mice were subcutaneously inoculated with A549 cells in the right flank, after the tumor volume was about 50 mm^3^. Mice were randomly separated into 3 groups, and acacetin was injected at 0, 10, and 20 mg/kg body weight every other day, and the tumor volume was recorded. (**B**, **C**) pictures of (**B**) and the weight of (**C**) tumor tissues isolated from mice after the last treatment (student t-test, **, *p* < 0.01; ***, *p* < 0.001; compared with 0 mg/kg group). (**D**) Western blotting results for apoptosis-related proteins, and cell cycle arrest-related proteins of tumor tissues (GAPDH was used as internal control for western blotting).

Next, we investigated the effects of acacetin on the A549-xenografted tumors. As shown in Fig. 3A–C, acacetin significantly suppressed the formation of A549-xenograft tumors in a dose-dependent manner, while no significant major organ toxicities were observed at the end of the experiment (Supplmentary Fig. 1). Afterward, the levels of apoptotic proteins and cell cycle-related proteins were detected by western blotting of A549-tumors. It was determined that acacetin dramatically induced the levels of Bax and Bak, while it suppressed the levels of Bcl-2; and, at the same time, both cyclinB1 and cyclin D were decreased after acacetin treatment (Fig. 3D).

### Acacetin upregulated expression of p53, miR-34a and downregulated expression of PD-L1 of NSCLC in vitro and in vivo

Later, we investigate the molecular mechanisms of the biological effects of acacetin on NSCLC cells.

**Figure 4:**
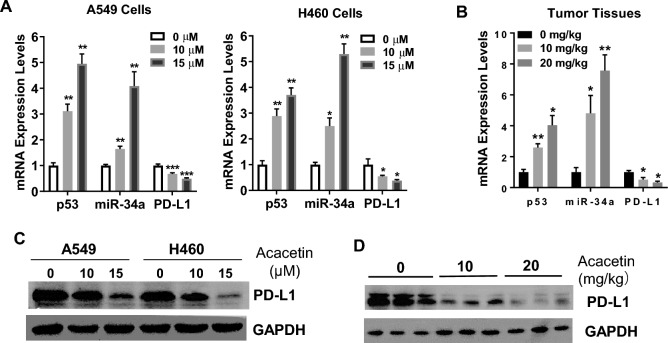
Acacetin upregulated expression levels of p53 and miR-34a, while downregulated expression levels of PD-L1 of NSCLC in vitro and in vivo. (**A**, **B**) A549 and H460 cells were treated 0, 10, and 15 μM of acacetin for 48 h, mRNA levels of p53, miR-34a and PD-L1 were detected by Q-PCR (**A**), and protein levels of PD-L1 were detected by western blotting (**B**). (**C**, **D**) mRNA levels of p53, miR-34a and PD-L1 were detected by Q-PCR (**C**), and protein levels of PD-L1 were detected by western blotting (**D**) of A54-Xenograft tumor tissues. (*, *p* < 0.05; **, *p* < 0.01; ***, *p* < 0.001, compared with 0 μM group or 0 mg/kg group; GAPDH was used as an internal control for western blotting).

P53 is a well-known tumor suppressor^19^, and acacetin inhibited cancer cell activities by inducing the expression of p53 in various types of cancer cells^20–22^. We found that the levels of p53 were significantly upregulated in acacetin-treated A549 and H460 cells in a dose-dependent manner (Fig. 4A). In vivo, the tumor levels of p53 in acacetin-treated A549-xenografted mice had significant increased, compared with untreated mice (Fig. 4B).

We then investigated the downstream targets of p53. Several studies have shown that p53 suppresses tumor cell viability by regulating the expression levels of microRNAs^23^. It has been shown that miR-34a is a direct target of p53^24^ and is one of the key mediators that inhibit NSCLC growth^25^. Firstly, we checked the changes of miR-34a expression levels after acacetin treatment in A549 and H460 cells. Acacetin significantly induced the expression levels of miR-34a in both NSCLC cells in a dose-dependent manner, compared with control (0 μM) group (Fig. 4A). In vivo, acacetin also dramatically induced miR-34a expression of tumor tissues in a dose dependent manner in A549-xenografted mice (Fig. 4B).

PD-L1 is a target of miR-34a, and plays an important role in progression and invasion of NSCLC^13^. We found that acacetin could significantly suppress the mRNA levels of PD-L1 both in NSCLC cells and in A549-xenografted tumor tissues in an acacetin dose-dependent manner (Fig. 4A,B). For protein changes of PD-L1, 15 μM of acacetin in vitro (Fig. 4C) and 20 mg/kg of acacetin in vivo (Fig. 4D) drastically inhibited the protein levels of PD-L1 of NSCLC cells and A549-xenografted tumor tissues, respectively. These results indicate that miR-34a and its target PD-L1 may play an important role in acacetin-induced NSCLC suppression.

**Figure 5:**
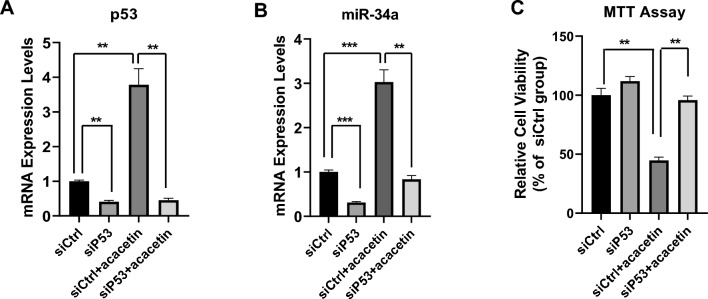
Knockdown of p53 reversed the induction effects of acacetin on miR-34a expression and abolished the inhibitory activity of acacetin on cell proliferation in A549 cells. A549 cells were transfected with control siRNA (siCtrl) or p53 siRNA (siP53) for 24 h, then cells were treated 0 μM or 15 μM of acacetin for 48 h, (A & B): mRNA expression levels of p53 (**A**) and miR-34 (**B**) of each group were detected by Q-PCR; (**C**) cell viability of each group were detected by MTT assay. (*, *p* < 0.05; **, *p* < 0.01; ***, *p* < 0.001, compared with siCtrl group).

To investigate whether acacetin-induced upregulation of miR-34a is mediated by p53, we suppressed the expression of p53 using specific siRNAs. As shown in Fig. 5, compared with the control group (siCtrl), in p53-siRNA group (siP53), the expression levels of p53 and miR-34a were reduced by almost 60% (Fig. 5A) and by 70% (Fig. 5B), respectively. At the same time, we found that acacetin did not induce the expression of p53 (Fig. 5A) and acacetin-induced upregulation of miR-34a was drastically inhibited by p53-knockdown in A549 cells (Fig. 5B). In addition, MTT assay data showed that the inhibition effects of acacetin on A549 cell proliferation were abolished when p53 expression levels had knocked down (Fig. 5C).

Overall, this data suggests that p53 plays crucial roles in the biological activities of acacetin on NSCLC cells. Acacetin induces the expression of miR-34a by upregulating p53, and acacetin suppresses NSCLC cell proliferation via regulating p53/miR-34a signaling.

### miR-34a mediated the anti-tumor effects of acacetin on NSCLC cells in vitro and in vivo

**Figure 6:**
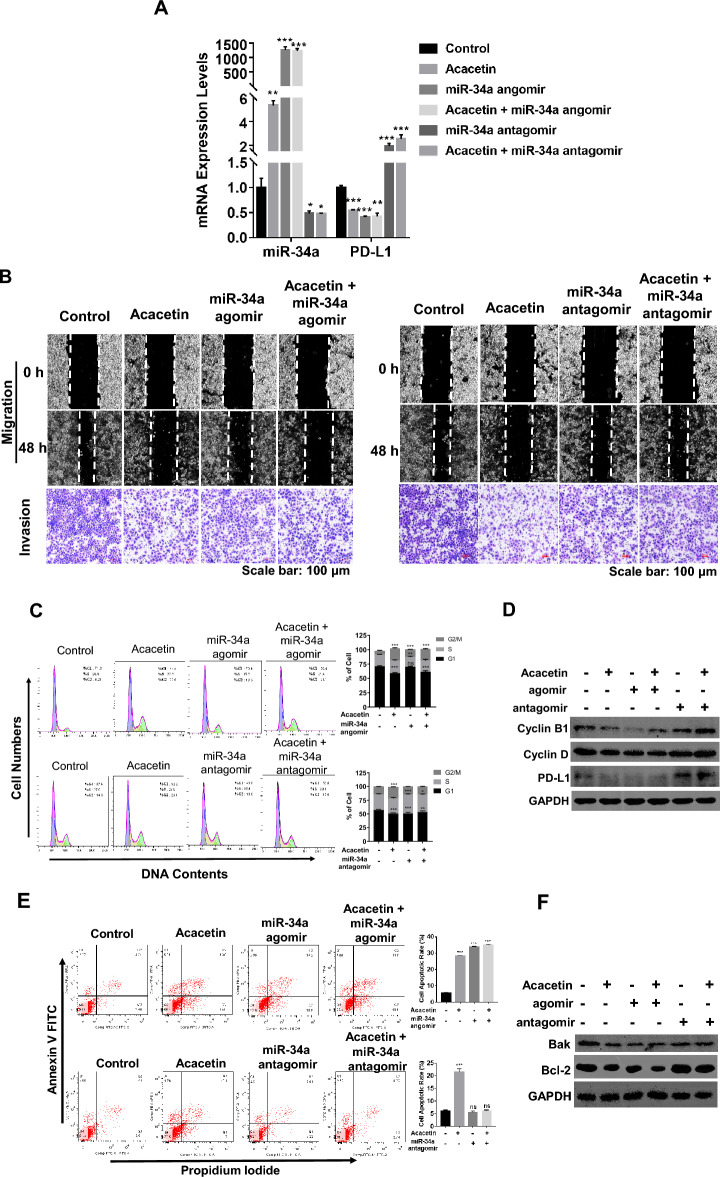
miR-34a mediated acacetin’s effects on NSCLC cells in vitro*.* A549 cells were transfected with miR-34a agomir, and antagomir, or their negative controls (NCs), then cells were treated with15 μM of acacetin for 48 h, (**A**) the expression levels of miR-34a of each group were detected by Q-PCR. (**B**) Cell migration and invasion assays for miR-34a agomir/antagomir-transfected or NC-transfected cells, which were treated with or without acacetin. (**C**) Cell cycle phases were analyzed by flow cytometry, and the histogram graphs shown are representative of three parallel and independent experiments. (**D**) Western blotting results for cell cycle-related proteins, cyclin B1, cyclin D, and PD-L1, of A549 cells (GAPDH was used as internal control for western blotting). (**E**) Cells were stained with Annexin V-FITC and PI, and apoptotic rate were analyzed, and the histogram graphs shown are representative of three parallel and independent experiments (***, *p* < 0.001; ns, no significant difference; compared with 0 μM group). (**F**) Western blotting results for apoptosis-related proteins, Bcl-2 and Bak, of A549 cells (GAPDH was used as internal control for western blotting). (*, *p* < 0.05; **, *p* < 0.01; ***, *p* < 0.001, compared with control group).

Next, we investigated whether miR-34a is a key factor that mediates the anti-tumor effects of acacetin in NSCLC cells. A549 cells were transfected with miR-34a agomir or antagomir. Afterward, cells were treated with 5 μM of acacetin for 48 h. As shown in Fig. 6A, the mRNA levels of miR-34a had drastically been upregulated by agomir treatment, while the miR-34a antagomir significantly suppressed the expression levels of miR-34a, compared with the control group.

We next checked the effects of miR-34a agomir/antagomir on acacetin-induced suppression of NSCLC cell migration and invasion. As shown in Fig. 6B, miR-34a agomir alone also suppresses the migration and invasion. However, co-treatment with acacetin and miR-34a agomir on A549 cells showed similar effects, compared with acacetin treatment group; whereas, miR-34a antagomir completely inhibited acacetin-induced suppression of migration and partially inhibited acacetin-induced suppression of invasion of A549 cells (Fig. 6B). This data indicates that overexpression of miR-34a cannot enhance the effects of acacetin, while downregulation of miR-34a could only partially suppress the inhibiting effects of acacetin on the migration and invasion of A549 cells.

Next, we performed cell cycle analyses, as shown in Fig. 6C. Overexpression of miR-34a by agomir can significantly induce G2/M phase increase and S phase decrease, while having no effect on the G1 phase of the A549 cell cycle. However, compared with acacetin-treatment group, the co-treatment of acacetin and miR-34a agomir did not further induce the increase of G2/M phase of A549 cell cycle. When the expression of miR-34a was inhibited by antagomir, acacetin could not induce G2/M phase arrest of A549 cells (Fig. 6C), which was accompanied by the recovery of the expression levels of cyclinB1, Cyclin D, and PD-L1 (Fig. 6D). This data indicates that miR-34a plays an important role in acacetin-induced G2/M phase arrest of NSCLC cells.

Furthermore, we checked the functions of miR-34a on NSCLC cell apoptosis. Here, we found that while miR-34a angomir showed little effects, miR-34a antagomir dramatically alleviated acacetin-induced apoptotic rate, compared with the acacetin-treatment group, in A549 cells (Fig. 6E). We also checked the protein levels of Bak and Bcl-2, and found that, compared with acacetin-treatment group, miR-34a agomir had no effect on the protein levels of both proteins, whereas protein levels of Bcl-2 had not changed in the miR-34a antagomir group, compared with the control (untreated) group (Fig. 6F) in A549 cells, indicating that inhibition of miR-34a expression could abolish the apoptotic-effects of acacetin in NSCLC cells.

**Figure 7:**
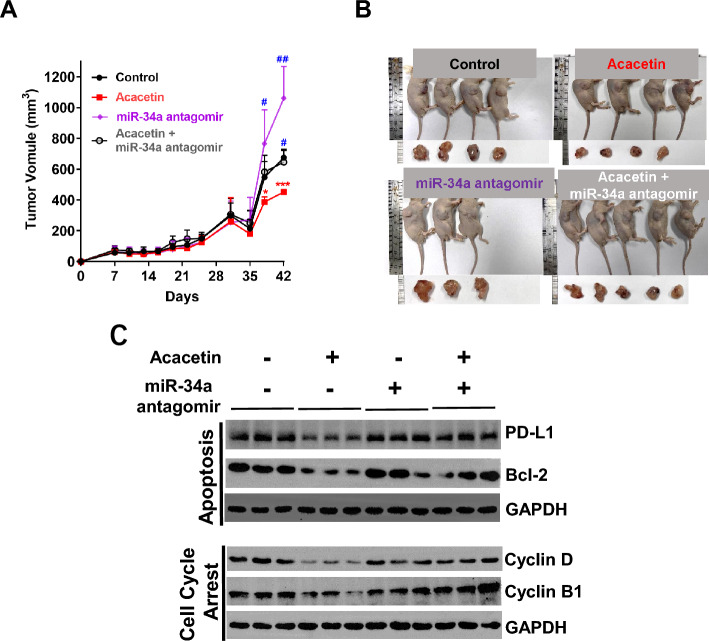
Acacetin showed no effects on the formation of A549-Xenogragted tumors when miR-34a was inhibited in vivo. Mice were subcutaneously inoculated with A549 cells in the right flank, and after the tumor volume had reached to about 50 mm^3^, mice were randomly separated into 6 groups, tumors were injected with miR-34a antagomir, or their negative control (NC) 5 times (on day 0, 7, 14, 21, 28, respectively), meanwhile, mice were treated with or without 20 mg/kg body weight of acacetin every other day, (**A**, **B**) The changes of tumor volume (**A**) and the pictures of mice and isolated tumors from mice after the last treatment (**B**). (**C**) Western blotting results for related proteins of tumors. (Student t-test, *, *p* < 0.05; ***, *p* < 0.001, compared with control group; #, *p* < 0.05; ##, *p* < 0.01, compared with acacetin group; GAPDH was used as an internal control for western blotting).

To further confirm the roles of miR-34a on the anti-NSCLC cell activity of acacetin in vivo, A549 cells were subcutaneously inoculated into BALB/c cells, and then were injected with miR-34a antagomir 5 times and mice were also treated with acacetin every other day. As shown in Fig. 7A, the tumor volume between acacetin group and antagomir + acacetin group appeared significantly different from day 35. The volumes of tumors that grew from the acacetin and miR-34a antagomir group were larger than those that grew from the acacetin group (Fig. 7B), indicating that downregulation of miR-34a expression inhibited the anti-tumor formation of acacetin on A549-xenografted mice. Furthermore, results from western blotting showed that miR-34a antagomir treatment could promote the expression of PD-L1 and Bcl-2 (Fig. 7C); and tumors of miR-34 antagomir treatment group showed higher protein levels of cyclin D and cyclin B1, compared with acacetin treatment group (Fig. 7C). This data indicates that miR-34a partially mediated the suppression of acacetin-induced xenograft tumor formation.

## Discussion

More and more evidence has shown that acacetin is a potent anti-cancer agent. Early on, though in vitro* analysis*, Hsu et al.^20^ found that acacetin inhibited A549 cells proliferation by suppressing G1/S phase of cell cycle progression by upregulating the levels of p53 and p21. Punia et al.^26^ demonstrated that acacetin could enhance the sensitivity of doxorubicin on NSCLC cells by synergistically inducing G2/M phase arrest and decreased clonogenic potential of NSCLC cells in vitro. In our study, using the two NSCLC cell lines, A549 and H460, acacetin was found to inhibit cell proliferation and apoptosis in a dose-dependent manner. Simultaneously, acacetin induced G2/M phase cell cycle arrest by inhibiting the expression levels of cyclin B1, the key regulator of cell cycle transition from G2 phase to M phase. In vivo, we found that acacetin dramatically suppressed A549-xenografted tumor formation in nude mice, and inhibited the expression levels of cyclin B1 and cyclin D. As cyclin D is a key regulator of G1/S phase transmission, a decrease of this protein may be related to the G1/S arrest. However, our results showed that acacetin had no effect on regulation of G1/S phase. Notably, recent studies showed that cyclin D plays an important role in invasion and metastasis of cancer cells^27^, and as our results also showed that acacetin inhibited invasion and migration of cancer cells in vitro, this may be partially mediated by the decrease in the levels of cyclin D protein.

One of the major anti-cancer effects of acacetin is to induce apoptosis of cancer cells by regulating different apoptotic-related molecules and signaling pathways in different cancer cells^4,5^. Acacetin induced apoptosis by activation of caspase cascades in prostate, gastric carcinoma, T cell leukemia, hepatocellular carcinoma, and head and neck squamous cell carcinoma cells^3^. At the same time, acacetin possibly induces apoptosis by inducing the accumulation of cellular levels of ROS in gastric carcinoma, and osteosarcoma cells^5,21^. Moreover, studies also found that acacetin could induce a decrease of mitochondrial membrane potential in cancer cells, which is accompanied by the changes of protein levels of bcl-2, bax, and bak, and in turn induces apoptosis of cells^4^. In this study, we found that the protein levels of bcl-2 were dramatically decreased after acacetin treatment, while the expression levels of apoptosis-promoter protein Bak were increased in an acacetin dose-dependent manner in NSCLC cells and in A549-xenografted tumors.

Previous studies found that acacetin induces apoptosis or suppression of cancer cell proliferation via p53 related pathways in various types of cancer cells, such as in hepatocarcinoma tumor, lung cancer, and gastric cancer cells^20–22^. We also found that acacetin dramatically induced the expression levels of p53 in NSCLC cells and in A549-xenografted tumor tissues.

Several studies have shown that polyphenols are potent chemopreventive agents for cancer treatment by targeting microRNAs. Polyphenol such as curcumin could possibly inhibit prostate cancer cell proliferation by inducing the expression of miR-34a^14^. Although mounting evidence shows that acacetin could inhibit various types of cancer cell proliferation, it is still unknown whether microRNAs are key players in this process. As miR-34a is a potential biomarker and a therapeutic target in cancer progression, we investigate whether acacetin regulates the expression levels of miR-34a. We found that, in vitro, acacetin could induce the expression levels of miR-34a, while miR-34a antagomir reverses acacetin-induced anti-proliferative effects in NSCLC cells. It is found that miR-34a is a direct target of p53^24^. We found that decreases of p53 reversed the induction effects of acacetin on miR-34a expression, while at the same time, the inhibitory effects of acacetin on the proliferation of NSCLC cells was abolished when p53 had decreased.

It has been demonstrated that miR-34a regulates cancer cell proliferation by targeting specific gene expressions, including PD-L1^28^. In NSCLC cells, the expression levels of PD-L1 were regulated by p53 via miR-34a^13^. We found that the expression levels of PD-L1 were downregulated after acacetin treatment, and miR-34a antagomir could reverse this effect of acacetin in vitro and in vivo, indicating acacetin-induced decreases of PD-L1 is in a miR-34a dependent manner. However, whether PD-L1 is one of the key factors on acacetin’s effects on NSCLC needs to be further investigated.

In conclusion, our findings determined new anti-tumor mechanisms of acacetin, and also found that acacetin induces p53 expression, which in turn regulates the expression of miR-34a, and that miR-34a is an important mediator on the anti-cancerous effects of acacetin in NSCLC.

## Materials and methods

### Chemicals and reagents

RPMI-1640 was purchased from Gibco, 2457402; Fetal bovine serum was purchased from Viva cell, 2209058; 3-^4,5-dimethylthiazol-2-yl^-2,5-diphenyl tetrazole bromide (MTT) was purchased from Suolarbio, M8180; Dimethyl sulfoxide (DMSO) was purchased from Mreda, M1538; Propidium iodide of cell cycle was purchased from Invitrogen, 2238888; RNase was purchased from ATRANS, GE101-01; The cell apoptosis kit was purchased from Suolarbio, CA1020; The matrigel basement membrane matrix was purchased from Corning, 356234; SDS-PAGE protein loading buffer (5×) was purchased from Beyotime, P0015L; Acacetin was purchased from MedChemExpress (MCE), HY-N0451.

### Cell culture

Human NSCLC cell lines, A549 and H460, were purchased from iCELL Bioscience (Shanghai, China) and were cultured in RPMI-1640 medium supplemented with 10% (v/v) fetal bovine serum, and 2 mM L-glutamine/100 U penicillin–streptomycin (Gibco) at 37 °C in a humidified 5% CO_2_ incubator as was previous reported^29^.

### Cell viability assay

Cell viability of cells was determined by MTT method. The cells were inoculated with 2.5 × 10^3^/well into the 96-well culture plate for attachment. Control group, and experimental group were set, with 6 multiple wells in each group. Cells were cultured overnight and were then treated with a DMSO (1%) solvent and varying concentrations of acacetin. They were Incubated with 5% CO_2_ at 37 °C for 24 h, or 48 h, 10 μL of MTT solution (5 mg/mL) was added to each well. After incubation for 4 h, the medium was removed, 150 μL DMSO was added to each well, and the absorbance was measured at 450 nm using an enzyme label.

### Cell cycle assay by flow cytometry

A549 cells and H460 cells were inoculated into 6-well plates at a density of 3.0 × 10^5^ cells/well and 2.5 × 10^5^ cells/well, respectively, were incubated at 37 °C for 12 h, and were then treated with different concentrations of acacetin. After incubating at 37 °C for 48 h, the cells were collected, and then fixed with 70% ethanol at − 20 °C overnight. The next day, the cells were centrifuged and resuspended with PBS, and were then stained with Propidium iodide (Invitrogen). Then the cell cycle was analyzed using flow cytometry and the cell cycle histogram was made using Graphpad prism software.

### Apoptosis assay by flow cytometry

A549 cells and H460 cells were inoculated into 6-well plates at a density of 3.0 × 10^5^ cells/well and 2.5 × 10^5^ cells/well, respectively, incubated at 37 °C for 12 h, and then were treated with different concentrations of acacetin. After 48 h, the cells were collected, and were treated according to the ANNEXIN V-FITC/PI Apoptosis Detection Kit instructions (Solarbio). Apoptosis was analyzed by flow cytometry and the early and late apoptosis rates were calculated. An apoptosis histogram was then made using Graphpad prism software.

### Wound healing assay

The migration ability of cells was determined by wound scratch method. The cells were inoculated into 12-well plates with 2.5 × 10^5^ cells/well. After cells were overgrown, a scratch was made by scratching on the bottom of the plate with a 10 μL pipette tip. Next, the old medium was replaced by a serum-free fresh medium with or without acacetin. Imaging was performed at 0 h, 24 h, and 48 h using a microscope.

### Invasion assay

Firstly, matrigel-transwells were generated. Matrigel (Corning, USA) was diluted at 1:9 ratio with a serum-free and antibiotic-free 1640 medium with a 24-well Transwell (JETBIOFIL) containing an 8 μm-well polyester film. 100 μL of diluted medium was added to each well and incubated at 37 °C for 4 h in a 5% CO_2_ incubator. Then, A549 and H460 cells were starved overnight and then diluted with serum-free medium with or without acacetin into the upper cavity of Transwell with 6 × 10^4^ cells/well and 8 × 10^4^ cells/well, respectively. 600 μL of serum-free medium with or without acacetin was added to the lower cavity. 48 h later, cells were fixed with 4% formaldehyde for 15 min, and then stained with 0.5% crystal violet for 30 min. Six visual fields were randomly selected under an inverted microscope, and the pictures were taken with microscope.

### Transfection

The control siRNA, p53-specific siRNAs, miR-34a agomir, antagomir, and their negative control (NC) sequences were designed and synthesized by Genepharma Co., Ltd (Shanghai, China).

The control siRNA (siCtrl) and p53 siRNAs (siP53) are as follows: siCtrl: 5′-UUC UCC GAA CGU GUC ACG UTT-3′; siP53: 5′-CUA CUU CCU GAA AAC AAC GTT-′3. The miR-34a agomir sequences (double stranded) are as follows: sense sequence 5′-UGG CAG UGU CUU AGC UGG UUG U-3′, antisense sequence 5′-AAC CAG CUA AGA CAC UGC AAU U-3′, while agomir NC sequences are: sence sequence, 5′-UUC UCC GAA CGU GUC AGG UTT-3′, antisense sequence 5′-ACG UGA CAC GUU CGG AGA ATT-3′. The miR-34a antagomir sequence (single stranded) is 5′-ACA ACC AGC UAA GAC ACU GCC A-3′, while its NC sequence is 5′-CAG UAC UUU UGU GUA GUA CAA-3′.

A549 or H460 cells were split into 6-well plate and were cultured. When the cells were 60–70% confluence, cells were then transfected with miR-34a agomir, antagomir, or their NCs using Lipofectamine 3000 transfection reagent (Invitrogen, Carlsbad, CA) under the suggestions of manufacturer’s manual. At 48 h after transfection, cells were collected for total RNA extraction and were further analyzed.

### Total RNA and protein extraction

#### Total RNA extraction

Total RNA was extracted by the Trizol method. Cells were lysed with 1 mL of Trizol, and the organic phase and water phase were separated by 200 μL of chloroform. After collecting the water phase, isopropyl alcohol was used to precipitate the RNA, 75% ethanol was used twice to clean the RNA, and RNA was eluted with 20 μL of RNase free water. RNA concentration was determined by Nanodrop ONE C (ThermoFisher Scientific).

#### Total protein extraction

A549 and H460 cells were collected and put on ice with lysis buffers (Tris–HCl pH-6.8, glycerin, 10% SDS, DTT, and protease inhibitors), and the cells were ultrasonically broken using a cell crusher. After centrifugation at 12,000 rpm at 4 °C for 10 min, the supernatant was collected and quantified using a Beyotime Biotechnology (BCA) protein extraction kit. The protein was stored in a refrigerator at -80℃ for later use.

### Real time quantitative PCR (RT-PCR) analyses

For miR-34a mRNA expression detection, the quantitative total RNA was reverse-transcripted into cDNA using a Mir-X miRNA First-Strand Synthesis Kit (Clontech, Takara Bio Inc., Dalian, China), and RT-PCR was performed using Mir-X miRNA qRT-PCR TB Green® Kit (Clontech, Takara Bio Inc., Dalian, China) according to the instructions provided in the kit manuals. U6 small nuclear RNA (snU6) was used as the endogenous miRNA control. Primers used for miRNA RT-PCR are as follows: miR-34a (forward primer: 5′-TGGCAGTGTCTTAGCTGGTTGT-3′; reverse primer: 5′-CGAATTCTAGA GCTCGAGGCAG-3′), U6 (forward primer: 5′-CGCAAATTCGTGAAGC GTTCC-3′; reverse primer: 5′-CGAATTCTAGAGCTCGAGGCAG-3′).

For the detection of p53 and PD-L1 mRNA expression, the quantitative total RNA was reverse-transcripted into cDNA using the FastKing cDNA first Strand Synthesis kit (Tiangen, KR116), and RT-PCR was performed according to the instructions of 2×TSINGKE® Master qPCR Mix (Cytology, TSE201), 18 s was used as internal control. Primers used are as follows: p53 (forward: 5′-CCTCAGCATCTTATCCGAGTGG-3′, reverse: 5′-TGGATGGTGGTACAGTC AGAGC-3′); PD-L1 (forward: 5′-TGCCGACTACAAGCGAATTACTG-3′, reverse: 5′-CTGCTTGTCCAGATGACTTCGG-3′); 18 s (forward: 5′-AAGTCCCTGC CCTTTGTACACA-3′, reverse: 5′-GATCCGAGGGCCTCACTAAAC-3′), which were synthesized by Beijing Cytology Biological Company.

The data was analyzed by 2^−△△Ct^ method to calculate the gene expression levels^30^, and the results were further analyzed using GraphPad Prism 5 software.

### Western blotting

Protein (30 μg/group) were separated with SDS-PAGE gel, and transferred to Polyvinylidene Difluoride (PVDF) membrane, followed by fixing with 5% skim milk and by incubating first antibodies at 4 °C overnight, and the second antibody at room temperature for 1 h. Finally, use a developer for imaging. First antibodies used in our study are anti-PD-L1 (1:1000), anti-b-cell lymphoma 2 (Bcl-2) (1:1000), anti-Bcl-2 related X protein Bax (1:1000), anti-Bad (1:1000), anti-cyclin D1 (1:1000), anti-Cyclin B (1:1000), and internal reference GAPDH (1:2000). Second antibodies are horseradish peroxidase labeled goat anti mouse IgG (H + L) (Beyotime, A0216), and horseradish peroxidase labeled goat anti rabbit IgG (H + L) (Beyotime, A0208).

### Animal experiments

All the animal procedures were reviewed and approved by the Animal Welfare Committee of the Southwest Medical University (Issue No. 20220801-011), and were performed in accordance with the animal care regulations of the Southwest Medical University and were in compliant with ARRIVE guidelines.

5–6-week old female BALB/c thymic nude mice (from Beijing HFK Bioscience Co., LTD) were used for animal experiments. All mice were housed in standard specific pathogen free (SPF) animal rooms at the Animal Service Center of the Southwest Medical University with a 12-h dark–light cycle (7 a.m.–7 p.m.) having free access to water and a regular chow diet. For experiments on the role of acacetin on A549-xenograft tumors, A549 cells were collected and were re-suspended with 150 μL of PBS (4 × 10^6^ cells). Cells were injected into the right flank of nude mice. The tumor size was measured using vernier calipers every three days. Length and width of tumors were recorded, and the volume of tumors was calculated by formulation V = (L × W^2^)/2 as previous reported^31^. After the volumes of tumors reached about 50 mm^3^, mice were randomly divided into three groups (n = 5). Next, three groups of mice were treated with 200 μL of vehicle solution (0.4% carboxymethylcellulose sodium and 0.9% NaCl), 10 mg/kg or 20 mg/kg acacetin in a 200 μL vehicle solution by oral gavage every other day for 5 weeks. The mice were then humanely euthanized by isoflurane followed by cervical dislocation, and tissues were collected for further analyses.

For experiments on the role of miR-34a in acacetin-inhibited tumor formation, 4 × 10^6^ of A549 cells in 150 μL PBS were injected into the right flank of nude mice. When the tumor volume had reached 50 mm^3^, mice were randomly divided into 4 groups (n = 5 for each group), two groups of mice were then treated with vehicle solution (200 μL/mouse), the other groups of mice were then treated with 20 mg/kg acacetin in 200 μL vehicle solution/mouse by gavages every other day. Each 2 groups of mice were injected with miR-34a antagomir (50 nmoL), or the negative controls (NCs) by intratumor injection, respectively, on 0, 7, 14, 21, 28 days after the first gavage of vehicle solution or acacetin. Tumor volumes and body weight were measured every 2 days. Finally, all mice were sacrificed, tumors were excised, and tumor weight was recorded. Tumors and major organs were collected for further analyses.

### Statistical analyses

The statistical analysis data was processed using GraphPad Prism 5.0 software and was expressed as mean ± standard deviation SD. The significant difference in experimental results was calculated using one-way ANOVA or student t-test to compare two or more groups, with each group having at least three independent experimental results. A p-value of less than 0.05 is represented as a statistically significant difference.

### Supplementary Information

Supplementary Information.

## Data Availability

The data presented in this study is available in the published article and are made available from the corresponding authors following reasonable request.
