# Supplementary material for: Acacetin inhibited non-small-cell lung cancer (NSCLC) cell growth via upregulating miR-34a in vitro and in vivo
Source: Sci Rep. 2024 Jan 29;14:2348. doi: 10.1038/s41598-024-52896-6 (PMC10824707; doi:10.1038/s41598-024-52896-6)

Supplementary Information

**Acacetin Inhibits Non-Small-Cell Lung Cancer (NSCLC) Cell Growth via Upregulating miR-34a *in vitro* and *in vivo*.**

**Jing Li^1,2,3^, Xianmei Zhong^3,4,5,6^, Yueshui Zhao^3,4,5^, Jing Shen^3,4,5^, Zhangang Xiao^3,4,5*^, Chalermchai Pilapong^1*^**

1. Laboratory of BioMolecular Imaging, Molecular and Cellular Biology, Department of Radiologic Technology, Faculty of Associated Medical Sciences, Chiang Mai University, Chiang Mai 50200, Thailand
2. Department of Oncology and Hematology, The Affiliated Traditional Chinese Medicine Hospital of Southwest Medical University, Luzhou 646000, China
3. Laboratory of Molecular Pharmacology, Department of Pharmacology, School of Pharmacy, Southwest Medical University, Luzhou 646000, China
4. Cell Therapy & Cell Drugs of Luzhou Key Laboratory, Southwest Medical University, Luzhou 646000, China
5. South Sichuan Institute of Translational Medicine, Luzhou 646000, China
6. Department of Pharmacy, People’s Hospital of Nanbu County, Nanchong 637300, China

* Authors to whom correspondence should be addressed.

Correspondence e-mails:

[chalermchai.pilapong@cmu.ac.th](mailto:chalermchai.pilapong@cmu.ac.th); [zhangangxiao@swmu.edu.cn](mailto:zhangangxiao@swmu.edu.cn)

**Materials and Methods**

**ALT & AST Assay**

ALT and AST detection kits were purchased from Nanjing Jiancheng Bioengineering Institute (Cat #: C009-2-1 and C010-2-1, respectively. Nanjing, China). Blood of the mice was collected at the end of the animal experiment, then the whole blood was centrifuged by 13,000 g for 5 min, transfer supernatant serum into new tube for ALT and AST assay. ALT and AST levels were detected following manufacturers’instructions.

**Hematoxylin and Eosin (H&E) Staining**

Liver, lung, kidney, and spleen of mice were collected, H&E staining were performed by Wuhan Servicebio Technology Co., LTD (Wuhan, China). Nuclei and basophilic cellular components are stained by blue color; Cytoplasm and acidophilic cellular components are stained by dark red color.

**Supplementary Figure S1:**

**
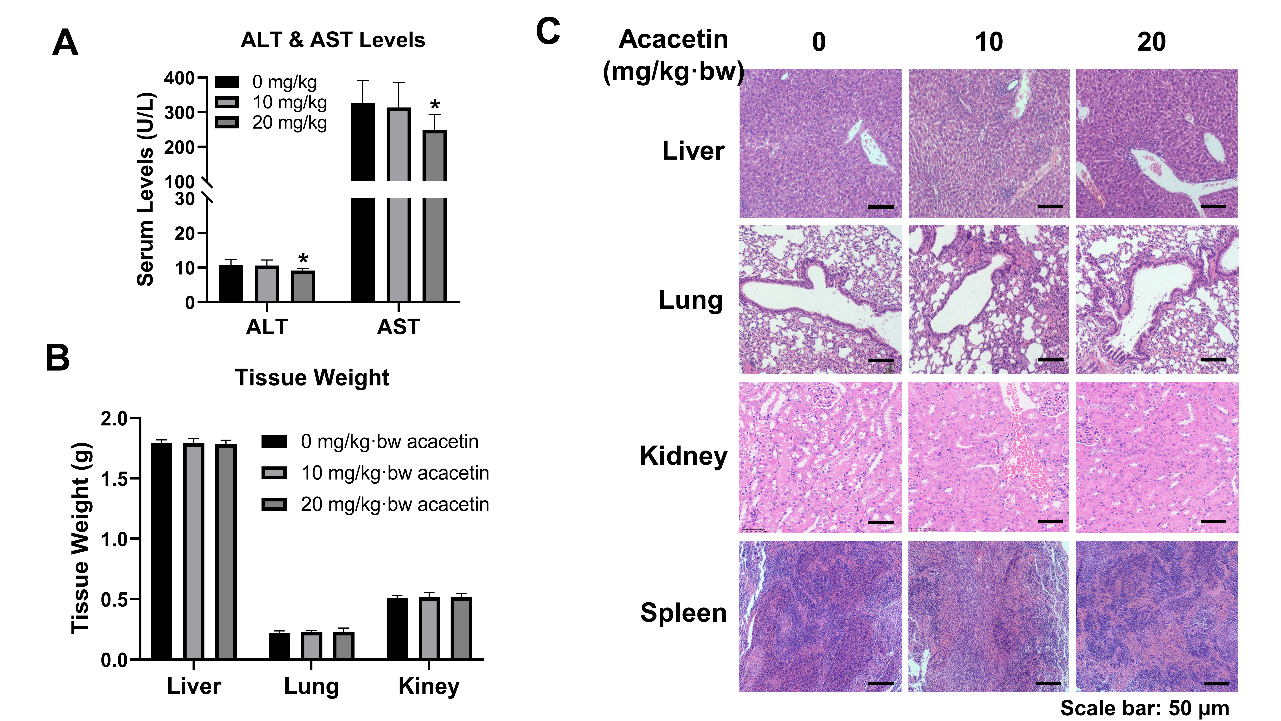
**

**Supplementary Figure S1: Acacetin showed no significant toxicities on major organs of A549-Xenografted mice.**

(A) Measurement of serum levels of ALT and AST; (B) Tissue weight of liver, lung, and kidney of each group; (C) H & E staining of liver, lung, kidney, and spleen (Nuclei and basophilic cellular components are stained by blue color; Cytoplasm and acidophilic cellular components are stained by dark red color). (*, p<0.05, compared with. 0 mg/kg bw acacetin group; scale bar: 50 μm)

**Supplementary Figure S2:**

**Original Figures for Blots in Figure 2**

**
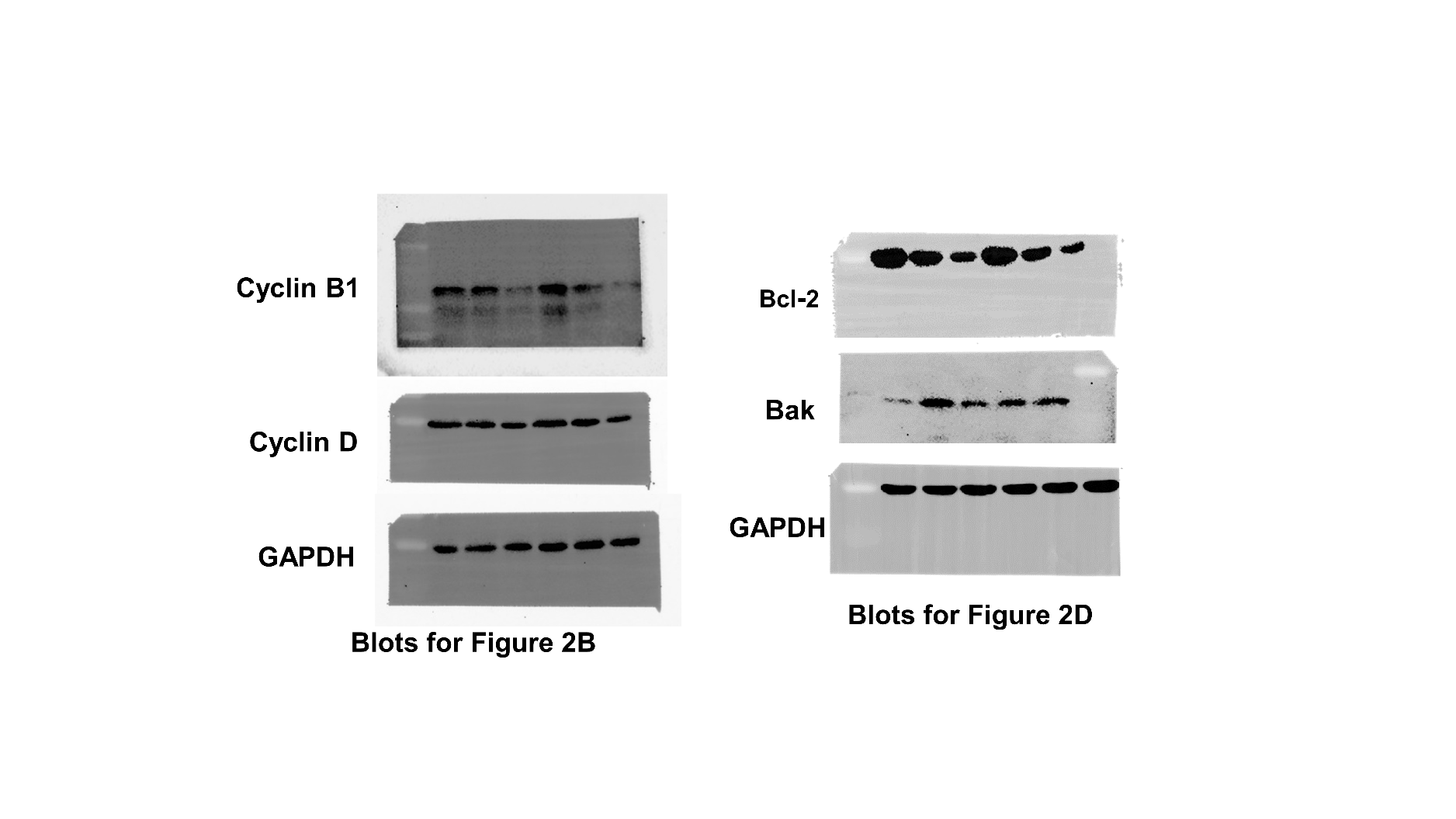
**

**Supplementary Figure S3:**

**Original Figures for Blots in Figure 3**


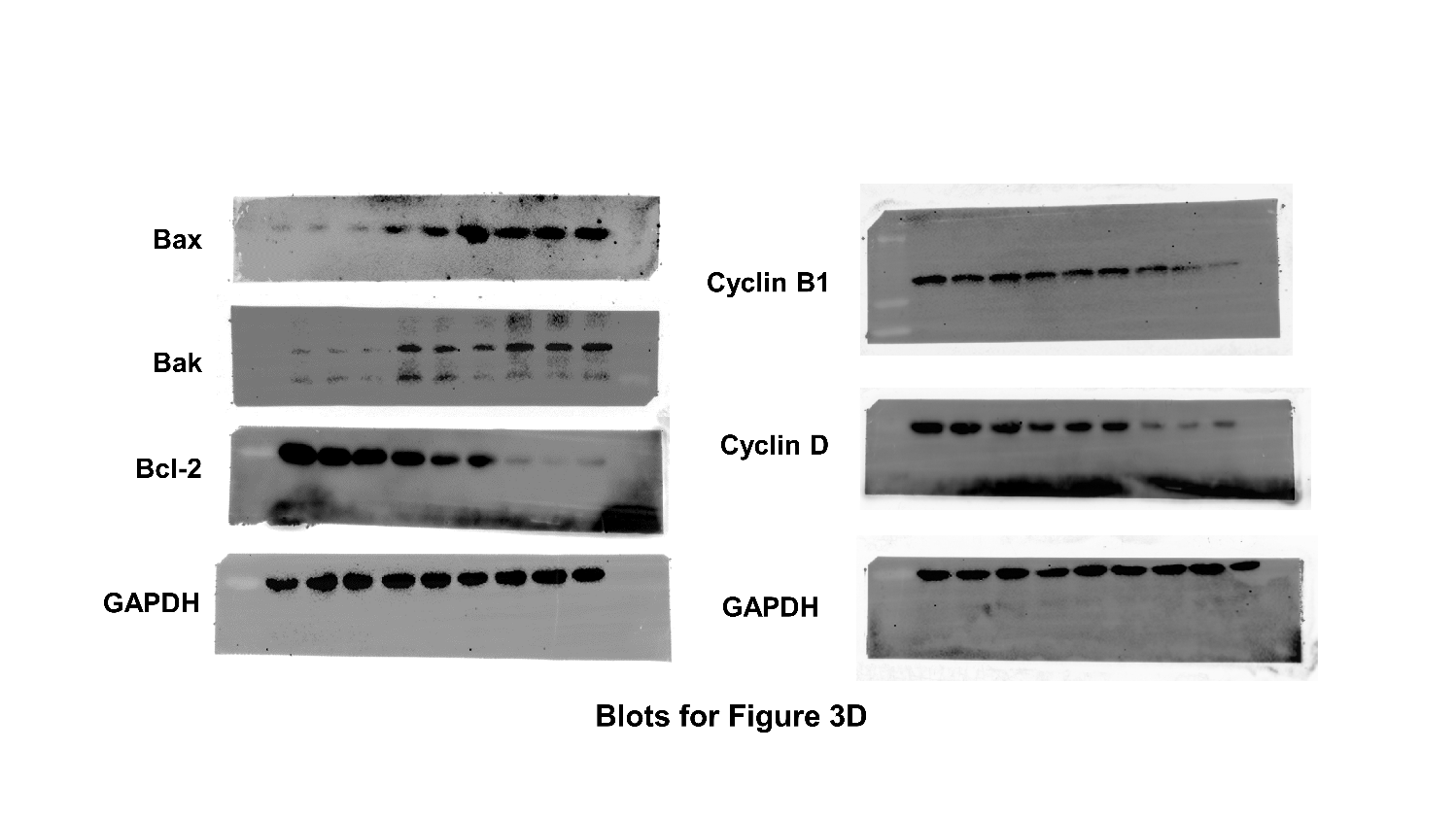


**Supplementary Figure S4:**

**Original Figures for Blots in Figure 4**


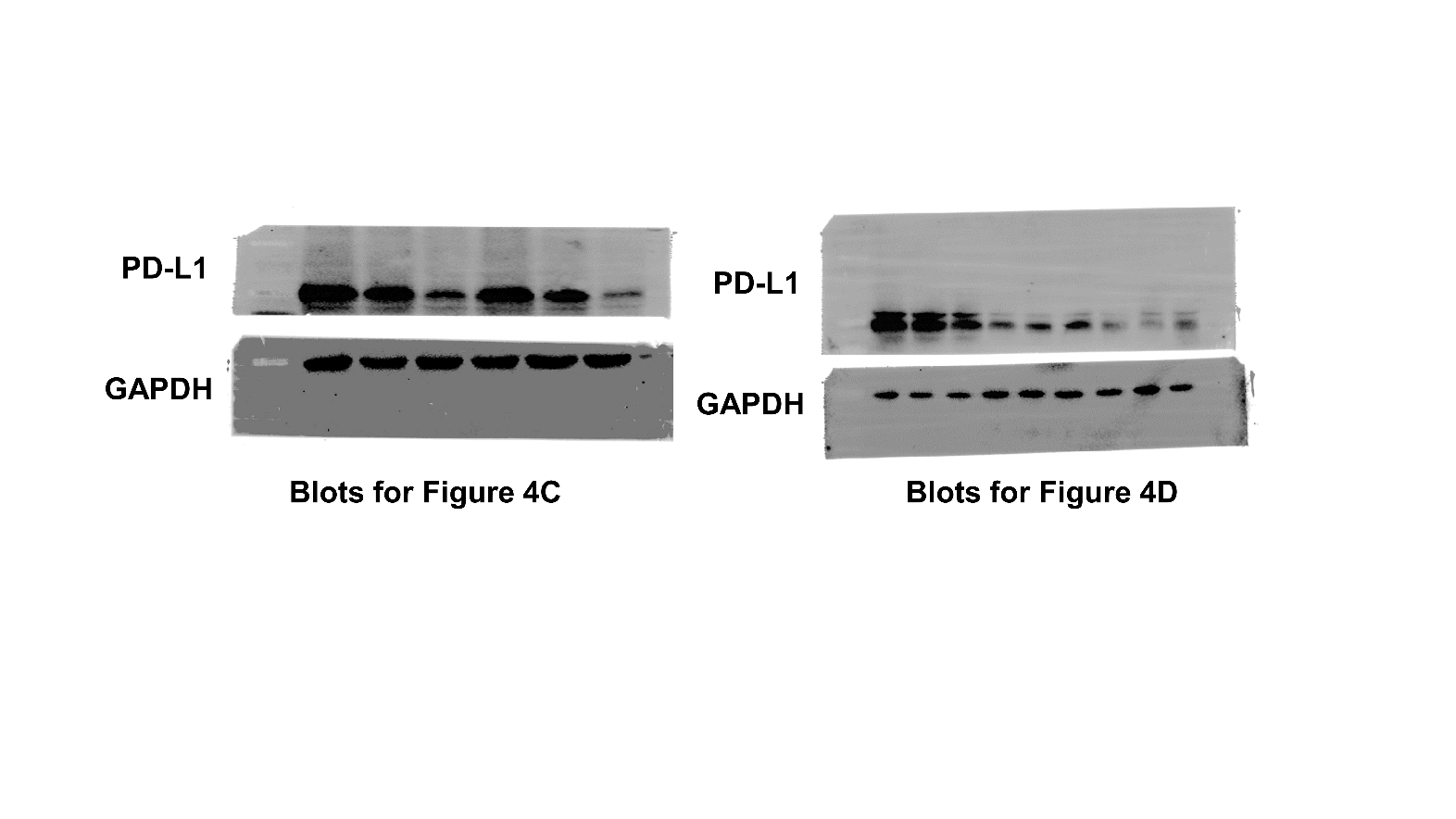


**Supplementary Figure S5:**

**Original Figures for Blots in Figure 5**


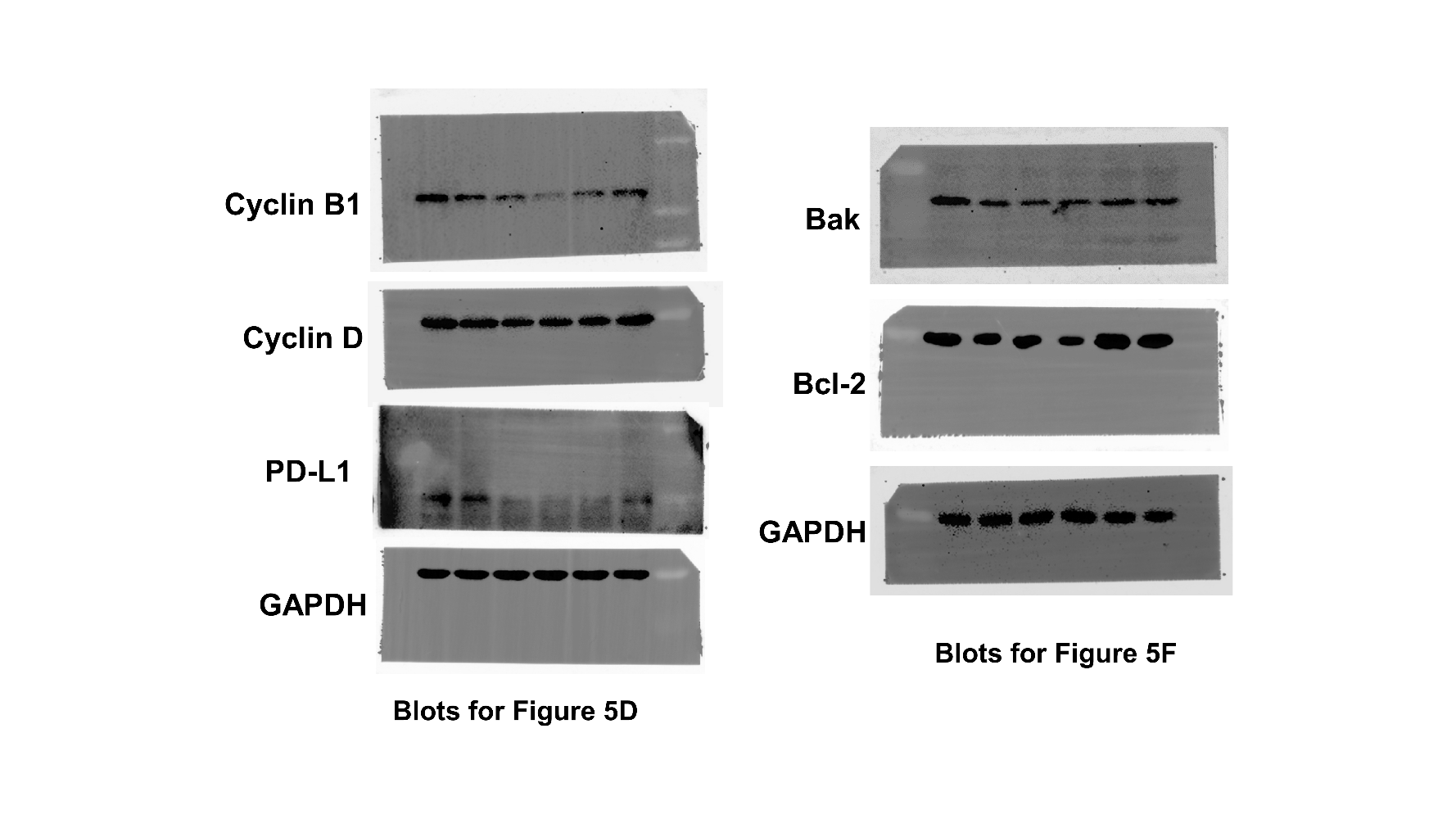


**Supplementary Figure S6:**

**Original Figures for Blots in Figure 6**


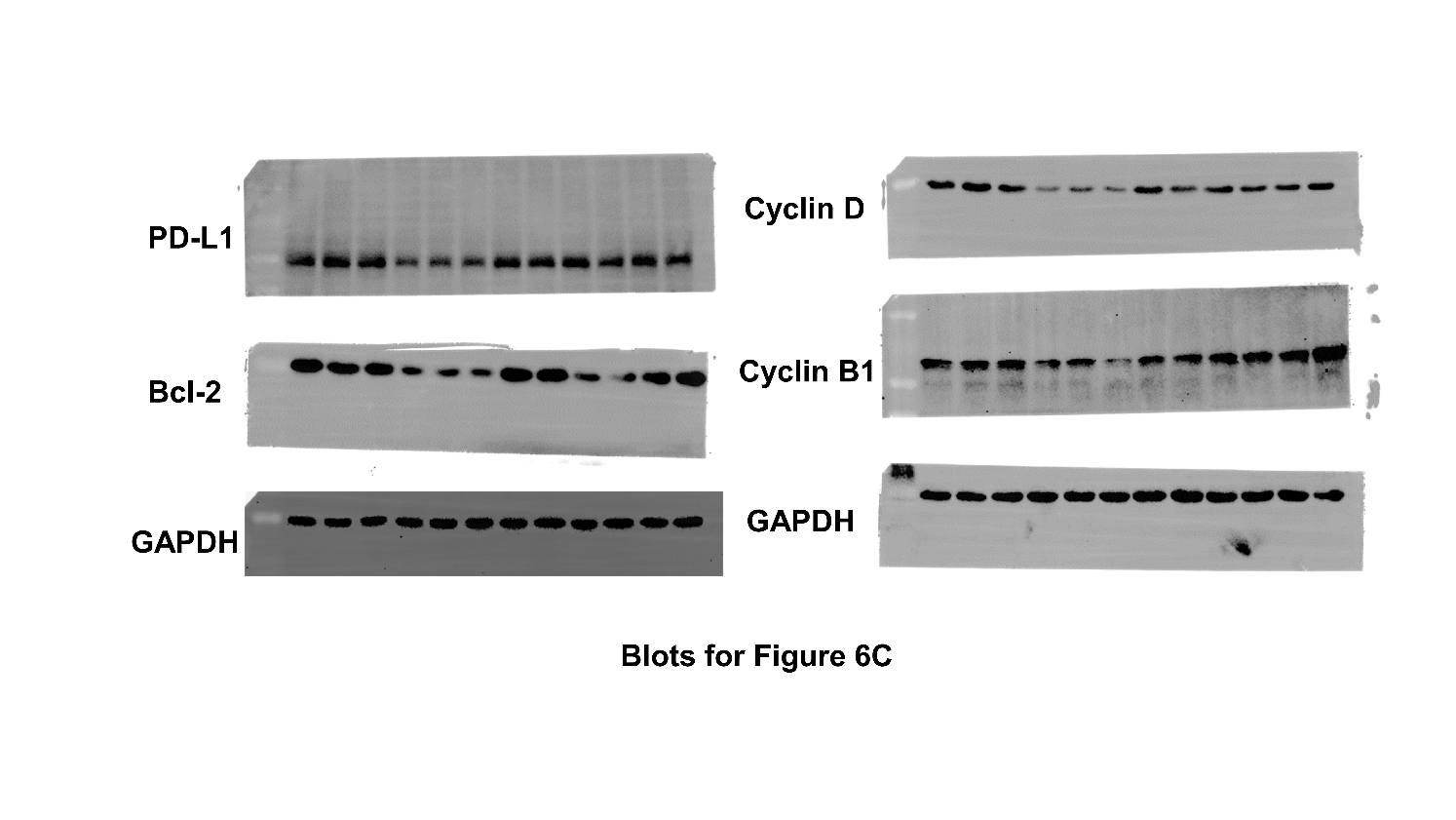

Supplement: Supplementary file 1 — Supplementary Information. [file 41598_2024_52896_MOESM1_ESM.docx]
